# Adult total wellness: group differences based on sitting time and physical activity level

**DOI:** 10.1186/1471-2458-14-234

**Published:** 2014-03-06

**Authors:** Faisal A Barwais, Thomas F Cuddihy, L Michaud Tomson

**Affiliations:** 1 School of Exercise and Nutrition Sciences, Institute of Health and Biomedical Innovation, Queensland University of Technology, Brisbane, Australia; 2 Department of Physical Education and Sports, Umm Al-Qura University, Makkah, Saudi Arabia; 3 School of Education and Professional Studies, Griffith University, Brisbane, Queensland, Australia

**Keywords:** IPAQ, The Wellness Evaluation of Lifestyle (WEL), Sedentary lifestyle

## Abstract

**Background:**

An increasing body of evidence associates a high level of sitting time with poor health outcomes. The benefits of moderate to vigorous-intensity physical activities to various aspects of health are now well documented; however, individuals may engage in moderate-intensity physical activity for at least 30 minutes on five or more days of the week and still exhibit a high level of sitting time. This purpose of this study was to examine differences in total wellness among adults relative to high/low levels of sitting time combined with insufficient/sufficient physical activity (PA). The construct of total wellness incorporates a holistic approach to the body, mind and spirit components of life, an approach which may be more encompassing than some definitions of health.

**Methods:**

Data were obtained from 226 adult respondents (27 ± 6 years), including 116 (51%) males and 110 (49%) females. Total PA and total sitting time were assessed with the International Physical Activity Questionnaire (IPAQ) (short-version). The Wellness Evaluation of Lifestyle Inventory was used to assess total wellness. An analysis of covariance (ANCOVA) was utilised to assess the effects of the sitting time/physical activity group on total wellness. A covariate was included to partial out the effects of age, sex and work status (student or employed). Cross-tabulations were used to show associations between the IPAQ derived high/low levels of sitting time with insufficient/sufficient PA and the three total wellness groups (i.e. high level of wellness, moderate wellness and wellness development needed).

**Results:**

The majority of the participants were located in the high total sitting time and sufficient PA group. There were statistical differences among the IPAQ groups for total wellness [*F* (2,220) = 32.5 (*p* <0.001)]. A Chi-square test revealed a significant difference in the distribution of the IPAQ categories within the classification of wellness [χ^2^ (*N* = 226) = 54.5, *p* < .001]. One-hundred percent (100%) of participants who self-rated as high total sitting time/insufficient PA were found in the wellness development needed group. In contrast, 72% of participants who were located in the low total sitting time/sufficient PA group were situated in the moderate wellness group.

**Conclusion:**

Many participants who meet the physical activity guidelines, in this sample, sit for longer periods of time than the median Australian sitting time. An understanding of the effects of the enhanced PA and reduced sitting time on total wellness can add to the development of public health initiatives.

## Background

Engaging in sufficient levels of moderate-to-vigorous intensity physical activities for at least 30 minutes on five or more days of the week has been shown to provide health benefits [1]. In the 21^st^ century, however, many children and adults around the world fail to engage in sufficient levels of activity required to derive such health benefits, and the research evidence suggests that PA levels worldwide may be still on the decline [2]. One possible contributor to this may be the increase in sedentary lifestyles at home, in the office and during leisure time [3]. This is largely related to the increasing popularity of computer usage, video gaming and television viewing. Increased levels of sedentary behaviors have been associated with decreased levels of PA [4].

Sedentary behavior (e.g., sitting, TV viewing, driving a car) refers to low energy expenditure behavior [1.0 to 1.5 metabolic equivalent units (METs), with 1 MET being energy expenditure at rest] and is distinct from physical inactivity, often conceptualized as a lack of moderate-to-vigorous PA [5]. Sitting time has been used as a specific marker of sedentary behaviors during waking hours [6,7]. Researchers [3] found that the average United States adult during the 2003-2004 period spent approximately 7.7 hours/day of their waking time engaged in sedentary behavior. Australian adults, on average, spent 9.3 hours/day of their waking time in sedentary activities [6].

An increasing body of evidence suggests that sedentary behaviors are associated with poor health outcomes such as obesity [8,9], Type 2 diabetes [10], high blood pressure [11], cardiovascular disease [12] and metabolic syndrome [13]. Despite the popularity of estimating sedentary behaviors and moderate and vigorous-intensity physical activities to determine the prevalence of, or relationships with, various aspects of health, there is little research to date that has explored the impact of sedentary behavior and of moderate- and vigorous-intensity physical activity on total wellness. The term “wellness” was first coined by Dunn, [14] and is defined as “an integrated method of functioning which is oriented toward maximizing the potential of which the individual is capable” (p.4). It is multi-faceted, involving six dimensions (i.e., physical, occupational, social, spiritual, intellectual, and emotional) that are enmeshed, related and, when balanced properly, provide the individual with optimal health or “high-level wellness” [15,16]. An individual can achieve optimal wellness through attending to each of the interconnected and dependent relationships among the dimensions of wellness [17,18] and changes in one dimension of wellness may affect other dimensions – in both positive and negative directions [19]. Thus, total wellness is a concept which encompasses much more than health-related perceptions [20].

The benefits of moderate and vigorous-intensity PA are numerous and affect all age groups, often in multiple dimensions of wellness [21-23]. In order to further explore this relationship, the purpose of this study was to examine differences in adult total wellness based on being in a group with high/low levels of sitting time combined with insufficient/sufficient PA. It was hypothesized that participants in the low level of sitting time group who engaged in sufficient PA would self-report levels of total wellness higher than those in the high level of sitting time with insufficient PA group.

## Methods

### Participants and procedures

Participants were recruited through email invitations, messages sent to registered members of a health institute and advertisements in the online student service news of a large, South East Queensland University, all of which included a link to a website. Ethics approval was deemed accepted by participants when they clicked the link and were connected to a Web page containing information about the study. It included access to the International Physical Activity Questionnaire (IPAQ) (http://www.ipaq.ki.se) and the Wellness Evaluation of Lifestyle (WEL) [24] as well as a consent form. Eligible participants were between the ages of 18 and 45 years. To proceed with the study, participants were instructed to click a button that indicated their consent to the online study. A convenience sample of 226 adults (mean age ± SD, 27 ± 6 years) volunteered to participate in this study, including 116 (51%) males and 110 (49%) females. Ethics approval was obtained from the University Human Research Ethics Committee at Queensland University of Technology (approval number UHREC 1100000358).

### Measurement

#### Physical activity

In order to assess to the level of physical activity recently achieved by the participants, the short version of the International Physical Activity Questionnaire (IPAQ) was used. This form contains objective questions regarding the frequency (days per week), duration (hours /minutes), level of intensity vigorous, moderate, walking of physical activity and sitting time during the last seven days. In a study which involved 12 countries, the IPAQ self-reported physical activity survey was shown to be reliable and valid [25]. The questionnaire was scored using established methods according to the IPAQ scoring protocol [26]. Total MET-minutes of physical activity were calculated by multiplying weekly physical activity volume (duration × frequency) of each activity by its corresponding MET value. Participants were categorized according to the American College of Sports Medicine (ACSM) guidelines established for adults: insufficiently active (participants who reported a PA level of 1-499 MET-minutes/week) and sufficiently active (participants who reported a PA level ≥ 500 MET-minutes/week) [27]. The time spent sitting during a usual weekday was considered a proxy measure of sedentary behavior. Participants were categorized as having high or low total sitting time with the cut-point being based on the median Australian value for total sitting time (240 minutes/day) reported by Bauman et al [28]. The membership of the high sitting time group was based on a sitting score ≥ 240 mins/day while the members of the low sitting time group had a score of ≤ 239 mins/day. Consequently, participants in this study were categorized into the following four groups: high total sitting time/insufficient PA; high total sitting time/sufficient PA; low total sitting time/insufficient PA and low total sitting time/sufficient PA.

### Wellness Evaluation of Lifestyle (WEL) Inventory

Wellness was measured using the online version of the WEL inventory, which was developed for institutions to simplify the collection and evaluation of data [29]. Derived from the Wheel of Wellness theoretical model, the WEL was developed as a method for describing wellness behaviors that encompass factors related to the participants body, mind, and spirit [24]. The Wheel model includes five major life tasks which are considered to be central to healthy functioning, are supported by empirical data and posit important characteristics of healthy persons. The WEL consists of 103 items represented as self-statements to which respondents reply using a five-point Likert- scale with the following options: (a) strongly agree, (b) agree, (c) undecided or neutral, (d) disagree and (e) strongly disagree. A score comprised of the 17 scales is computed by summing the 103 items and producing a total score (range = 103 to 515). For ease of interpretation, the total score is divided by the total points possible (515) to yield a percentage value. According to Myers et al. [30] total wellness percentages may be grouped into three categories: 1) 100% indicates a high level of wellness; 2) scores exceeding 80% indicate moderate wellness; and 3) scores below 79.9% indicate that there are areas in which further wellness development is needed [30].

Wellness may be seen as a dynamic construct which may change in different situations. It is rare and not “the norm” for people to a report total wellness score of 100%. People with a score of 80%, may be interpreted as indicative of moderate wellness. These people have room for improvement to reach greater wellness. Most people will report a total wellness score less than 79.9% [30]. Consequently, these people have life tasks which require further development. Low scoring life tasks are important and require further attention by the individual to assist them to attain the characteristics of healthy people [30]. The WEL has been shown to demonstrate construct validity and reliability in previous research [31] and has been used to assess wellness among adults [32].

### Statistical analyses

All statistical analyses were carried out using SPSS statistical software Version 21.0 for Windows (IBM SPSS Inc., Chicago, IL). To evaluate the effects of different amounts of sitting time and volume of physical activity on total wellness, an analysis of variance with descriptive statistics was implemented and the significance level was set at 0.05 for all tests. An analysis of covariance (ANCOVA) adjusting for age, sex and work status (student or employed**)** was used to determine if there were significant differences in total wellness scores among the four IPAQ-determined physical activity/sitting time classifications: high total sitting time/insufficient PA; high total sitting time/sufficient PA; low total sitting time/insufficient PA and low total sitting time/sufficient PA.

Cohen's d (i.e., difference in mean scores between groups divided by the pooled standard deviation) [33] effect sizes (ES) were calculated for the analysis of each variable to elucidate the meaningfulness of differences among physical activity level/sitting time classifications. Values for Cohen's *d* of 0.2, 0.5, and 0.8 were interpreted as small, moderate, and large, respectively [33]. Cross-tabulations were used to show associations between total wellness group membership (wellness development needed group and moderate wellness group) and IPAQ-determined physical activity/sitting time classification. The Chi-square test of goodness of fit was used to assess whether wellness grouping was likely to be associated with a specific IPAQ-determined physical activity/sitting time classification for the sample of 226 participants.

## Results

**Figure 1:**
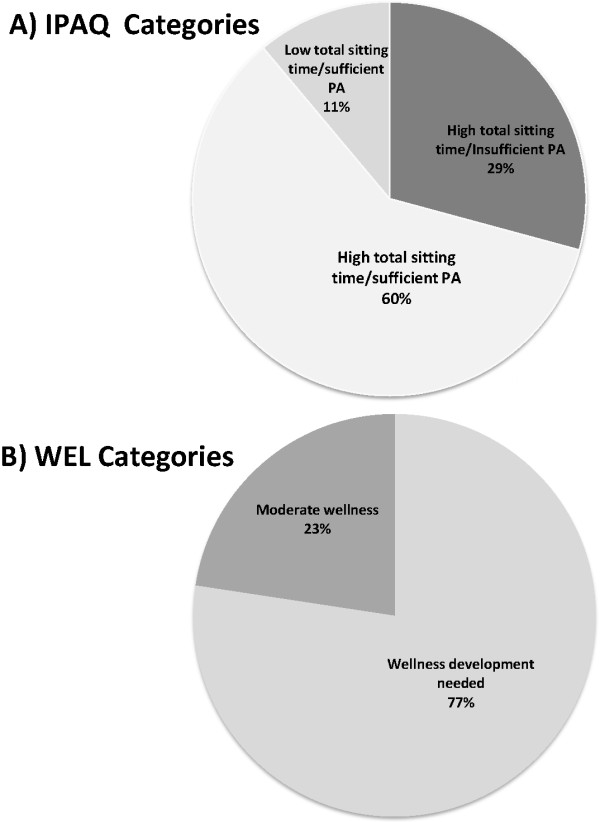
**The frequency of IPAQ and WEL scores. (A)** IPAQ constructed sitting time/physical activity categories (N = 226). **(B)** Wellness Evaluation of Lifestyle (WEL) categories (based on Total Wellness) (N = 226).

Participant characteristics are shown in Table 1. Frequency values indicate that there were more males (n = 116 26.1 ± 6), than females (n = 110 29.0 ± 7). The age range was from 19 to 43 years. Sixty-nine percent of participants were students, whereas 31% were employed. The frequency of IPAQ-determined physical activity/sitting time classifications is presented in Figure 1-A. The majority (60%) of the participants were categorized in the high total sitting time and sufficient PA group followed by 29% in the high total sitting time and insufficient PA group. No participants were found in the low total sitting time and insufficient PA category.

**Table 1:** General subject characteristics

	**Male**	**Female**	**Total**
Age (years)	(n = 116) 26.1 ± 6.1	(n = 110) 29.0 ± 6.8	(n = 226) 27.5 ± 6.6
18-25 years	(n = 79) 22.6 ± 1.5	(n = 48) 22.5 ± 1.5	(n = 127) 22.5 ± 1.5
26-35 years	(n = 20) 29.3 ± 2.7	(n = 35) 30.6 ± 2.9	(n = 55) 30.1 ± 2.9
36-45 years	(n = 17) 38.8 ± 2.3	(n = 27) 38.4 ± 2.0	(n = 44) 38.61 ± 2.1
Work status			
Student	(n = 98) 84% 24 ± 3.2	(n = 59) 53%% 23.4 ± 2.4	(n = 157) 69% 23.8 ± 3.0
Employed	(n = 18) 15% 37.7 ± 5.0	(n = 51) 46% 35.4 ± 3.8	(n = 69) 31% 36.0 ± 4.2

(Age mean ± SD).

Figure 1-B displays the frequency of WEL scores. The majority (77.4%) of the participants were categorized in the wellness development needed group followed by 22.6% in the moderate wellness group. No participants were found in a high level of wellness category.

**Figure 2:**
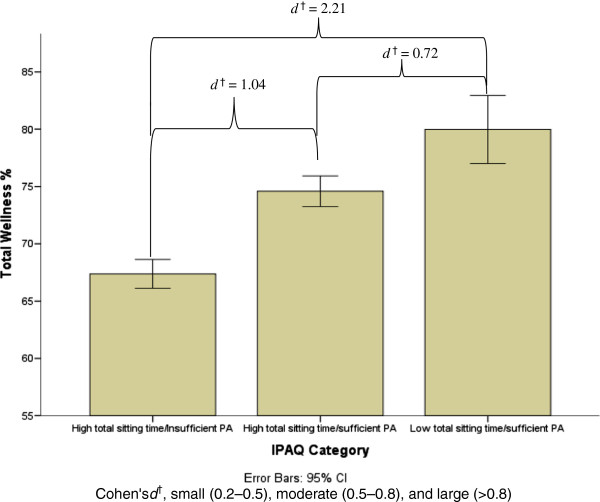
**Large and moderate differences in total wellness among IPAQ groups.** Mean plus error bars represent +/- 2 SE.

The analysis of covariance (ANCOVA) was used in order to determine whether significant differences in total wellness existed among IPAQ-determined physical activity/sitting time classifications. Results of the analyses indicate that, after adjustment for age, sex and work status (student or employed), there was a significant differences in total wellness scores among IPAQ-determined physical activity/sitting time groups [*F* (2,220) = 32.5 (*p* <0.001)]. Post-hoc tests showed these significant differences for total wellness to be between the high total sitting time/insufficient PA group (67%) and high total sitting time/sufficient PA group (74%) (*p* < .001) and between a high total sitting time/insufficient PA group (66.5%) and low total sitting time/sufficient PA group (80%) (*p* < .001). Moreover, a significant difference for total wellness was found between the low total sitting time/sufficient PA group (80%) and high total sitting time/sufficient PA group (74%) (*p* < .001) (Figure 2).

Large effect sizes were observed for differences in total wellness between the high total sitting time/insufficient PA and high total sitting time/sufficient PA groups (*d* = 1.04) and between the high total sitting time/insufficient PA and low total sitting time/sufficient PA groups (*d* = 2.21). A moderate effect size was observed (*d* = 0.72) for differences between the high total sitting time/sufficient PA and low total sitting time/sufficient PA groups (Figure 2).

Cross-tabulation of the two wellness groups (wellness development needed group and moderate wellness group) by the three IPAQ-determined physical activity/sitting time classifications is shown in Table 2. A Chi-square test revealed a significant difference in the distribution of IPAQ groups within the two wellness groups [χ^2^ (*N* = 226) = 54.4, *p* < .001]. One-hundred percent (100%) of participants who were in the IPAQ-determined high total sitting time/insufficient PA group were found in the wellness development needed group. By comparison, 72% of those in the IPAQ-determined low total sitting time/sufficient PA group, were located in the moderate wellness group, while the remainder of the participants were located in the wellness development needed group (28%). Of participants who were classified in the high total sitting time/sufficient PA group, 75% were in the wellness development needed group.

**Table 2:** Cross tabulation for sitting time and physical activity levels and total wellness

			**Classification of total wellness**		
			**Development needed**	**Moderate**	**Total**
**Physical activity levels**	High total sitting time/insufficient PA	Count	66	0	66
		% within IPAQ category	**100%**	0.0%	100%
	High total sitting time/sufficient PA	Count	102	33	121
		% within IPAQ category	**75.6%**	24.4%	100%
	Low total sitting time/sufficient PA	Count	7	18	25
		% within IPAQ category	28%	**72**%	100%
**Total**		Count	175	51	226
		% within IPAQ category	77.4%	22.6%	100%
		% within wellness group	100%	100%	100%

Chi-square (χ^2^ (*N* = 226) = 54.5, *p* < .001).

## Discussion

This study examined differences in total wellness among adults who self-reported high/low levels of sitting time and insufficient/sufficient PA. Results showed that more than half (60%) of participants were categorized in the high total sitting time/sufficient PA group. This finding provides support for the hypothesis that an individual can meet the public health recommendations for physical activity levels (30 min of moderate-intensity activity each day) yet still spend unhealthy amounts of time engaged in sedentary behaviors. These individuals are sometimes referred to as “active couch-potatoes” [34]. As hypothesised, those who spent less time in sitting and were sufficiently physically active were found in the moderate wellness group. Several studies have shown that participants from healthy populations who engage in higher physical activity levels may have higher levels of quality of life [35]. These higher levels are associated with improved physical and mental health, cognitive functioning, and social connectedness [36,37]. In contrast, participants who spent more time in sitting and were not sufficiently physically active were most likely to be found in the wellness development needed group. One recent study examined the influence of screen time (high or low) on health related quality of life across different levels of physical activity (none, insufficient or sufficient PA) among a large sample of Australian adults. Results indicated that adults who reported no PA (zero physical activity) in combination with high screen time were more likely to report lower health related quality of life [38]. Additional research found that individuals who were engaged in a sedentary lifestyle were significantly more likely to report poor health-related quality of life than other adults who engaged in moderate or vigorous physical activity [39]. As both high total sitting time and insufficient PA have been shown to have negative health effects [40,41], interventions should target a decrease of sedentary behavior as well as an increase of PA.

The findings of the current study are consistent with a Myers et al. [42] study of wellness that found no participants were in a high level of wellness category and the researchers suggested that high level wellness is not “the norm” in the United States. While this study employed a different wellness instrument, our findings are comparable to earlier studies, namely the association of total wellness with higher levels of physical activity. For example, Benzer and colleagues [43] conducted a study to explore the relationship between physical activity and perceived wellness, using the Perceived Wellness Survey (PWS) as the dependent variable. Greater levels of physical activity and leisure time activity were associated with higher perceived physical and psychological wellness scores. Those participants with a greater quantity of regular physical activity had greater overall perceived wellness scores. Those findings support the concept that an active lifestyle will be associated with greater overall wellness [43].

Wellness is a term that encompasses an individual’s outlook on life, including their perceptions of personal fitness, happiness, learning, society, work and spirituality [18]. According to the literature, wellness has many dimensions. Each dimension may be identified as having specific subscales and each is an integral part of the whole. Sedentary behavior and physical activity are subsumed within the physical dimension. The WEL model is based on the Adlerian theory that holism, an evaluation of the whole rather than the parts, was central to understanding human behavior. There is recognition that it is the interaction of the parts and the context in which a person lives that influences the whole [44]. Consistent with this philosophy, Myers and Sweeney [42] emphasized that all wellness dimensions are interrelated; a change in one area causes or contributes to changes to other areas, and this influences total wellness scores. The present study provides support for this notion, as participants who were engaged in low levels of sitting time/sufficient PA had significantly higher total wellness scores.

This research adds to the knowledge base by showing that sedentary behaviors (such as high sitting time and low physical activity) are not only associated with poor health outcomes [40,41] but also with the lowest wellness scores, which include more than health-related perceptions. In this sense, reducing sitting time and engaging in moderate or vigorous-intensity PA may be a valuable approach to improving total wellness. This information supports the endorsement of public health recommendations concerning PA for adults (i.e. all adults should accumulate 30 minutes of moderate intensity PA on most, preferably all, days during the week) y.

This study has several limitations. The IPAQ, which was used to measure sitting time and insufficient/sufficient PA in the current study, has some limitations, one of which is that only one question was used to measure time spent sitting. The IPAQ is not designed to provide a detailed assessment of sitting across daily life domains: (work, transportation, home and leisure time). Future studies would benefit by use of an instrument which targets sedentary behaviors, including sitting time, within these specific daily life domains. The role of sitting time may vary by home and leisure time domains, therefore, the key scientific questions of public health importance also relate to the quantity of a behavior as much as the context within which the behavior occurs. All responses in this study were self-reported, which may have resulted in biased conclusions. The current study was conducted through the Internet, thus, people could only respond to the IPAQ/WEL if they could access the Internet and were university students or alumni. Overall, this selection bias limits the generalizability of the results. Future studies should include larger sample sizes, more representative age ranges and more diverse education levels. Objective measures (e.g., accelerometer) of PA level and total wellness would also be beneficial.

## Conclusion

The results from this study provide meaningful information to enhance our understanding of the effects on total wellness of the combination of sitting time and insufficient/sufficient PA. One-hundred percent (100%) of participants in the high total sitting time/insufficient PA group were found in the wellness development needed group. Such knowledge is useful in arguing the importance of the development of public health initiatives that aim to increase PA and reduce sedentary behaviors such as sitting time. In addition, this research supports public health recommendations that target the “active couch potato” to identify that significant health benefits (and total wellness) may be achieved by adults who spend less time sitting as well as being sufficiently physically active.

## Competing interests

The authors declare that they have no competing interests.

## Authors’ contributions

FAB performed all work pertaining to this manuscript. TFC supervised the research, helped analyze the data and revised the manuscript. LMT participated in literature review, and revised the manuscript. All authors revised the text critically for important intellectual content and read and approved the final manuscript.

## Pre-publication history

The pre-publication history for this paper can be accessed here:

http://www.biomedcentral.com/1471-2458/14/234/prepub
